# Recurrent mandibular ameloblastoma in soft tissue and rib graft 17 years after initial presentation

**DOI:** 10.1186/s43046-019-0012-1

**Published:** 2020-01-06

**Authors:** Omar Hamdy, Sara Raafat, Gehad A. Saleh, Shadi Awny, Abdelhadi M. Shebl, Mohammed A. Hegazy

**Affiliations:** 1 Surgical Oncology Unit, Oncology Center Mansoura University (OCMU), Mansoura, Egypt; 2 Pathology Department, Faculty of Medicine, Mansoura University, Mansoura, Egypt; 3 Radiology Department, Faculty of Medicine, Mansoura University, Mansoura, Egypt

**Keywords:** Ameloblastoma, Recurrence, Autograft, Benign

## Abstract

**Background:**

Ameloblastoma is the commonest odontogenic tumour of epithelial origin with a high incidence for developing local recurrence. We present a patient who developed local recurrence in both soft tissue and bone graft 17 years after the initial presentation.

**Case presentation:**

A 75-year-old female with a previous history of right hemimandibulectomy and rib reconstruction for ameloblastoma in 1999 presented to our centre with a large cystic mouth floor swelling, biopsy from which revealed recurrent ameloblastoma. The patient underwent excision of the recurrent mass en bloc with the cystic swelling through oral and cervical approaches. The patient was discharged after 5 days with an uneventful postoperative course and with a free 2-year follow-up from further recurrence.

**Conclusion:**

Ameloblastoma is a locally aggressive tumour for which wide local excision with adequate margins is the best management approach. Recurrence of ameloblastoma even after adequate resection is not uncommon, and its management is considered a surgical challenge. A very long time may pass between the initial presentation and the development of recurrence.

## Background

Ameloblastoma is the commonest odontogenic tumour of epithelial origin with a high incidence for developing local recurrence even after excision with negative margins. Local recurrence can occur in the mandible, soft tissue and even in the reconstructive materials [1, 2]. We present a patient who developed local recurrence of ameloblastoma in both the soft tissue and the bone graft seventeen years after the initial presentation.

## Case presentation

**Fig. 1:**
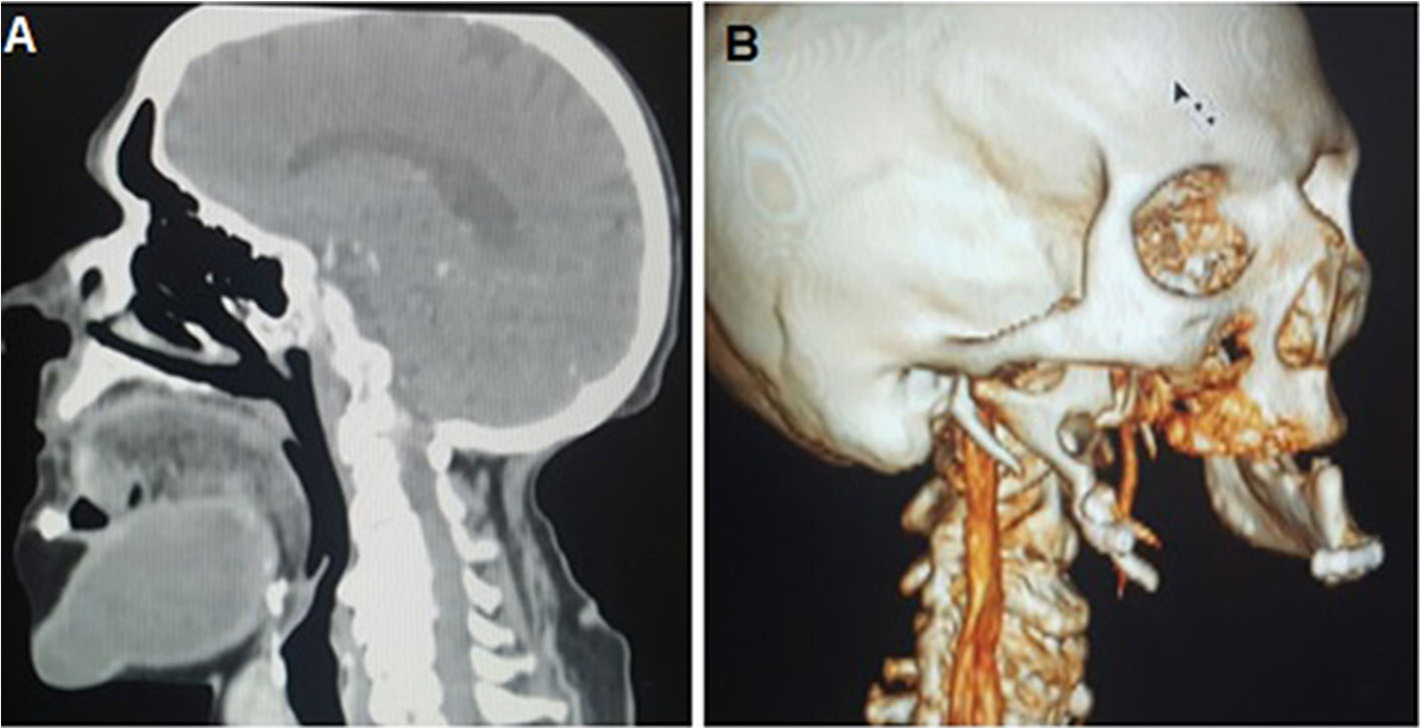
CT scan of the recurrence: Post-contrast sagittal reformatted CT image (**a**) shows large thick-walled necrotic cystic lesion at the right mandibular and submandibular regions with an erosion of the underlying bone graft. Volume rendering (VR) reconstructed CT image (**b**) confirms the destruction of the right-sided bone graft by the recurrent mass

**Fig. 2:**
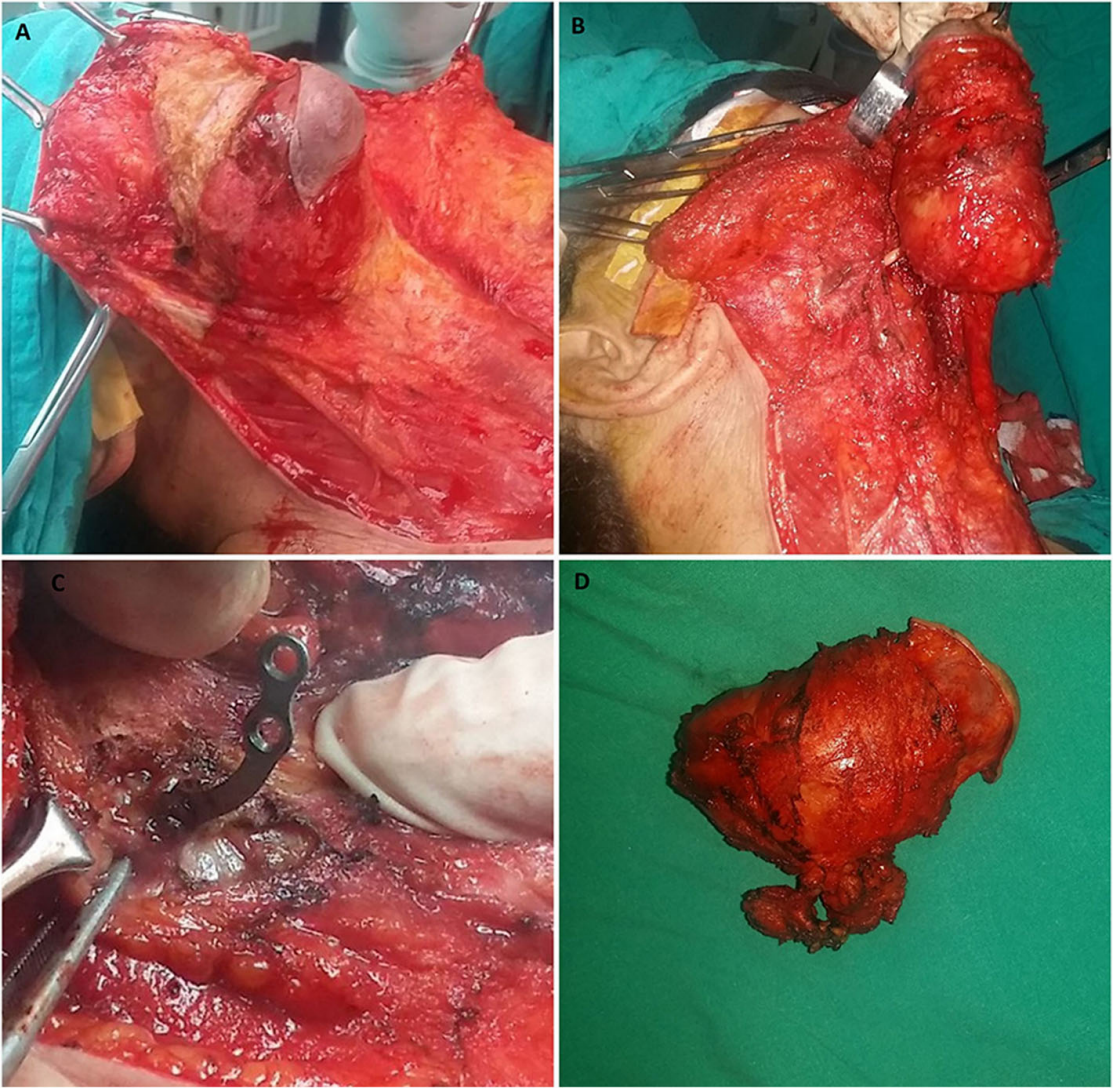
Operative photos. **a** After elevation of the flaps, the large cystic mandibular swelling is seen covered by a piece of skin. **b** After complete dissection of the swelling. **c** The non-toothed forceps holding tumour recurrence invading the metallic plate. **d** The surgical specimen

**Fig. 3:**
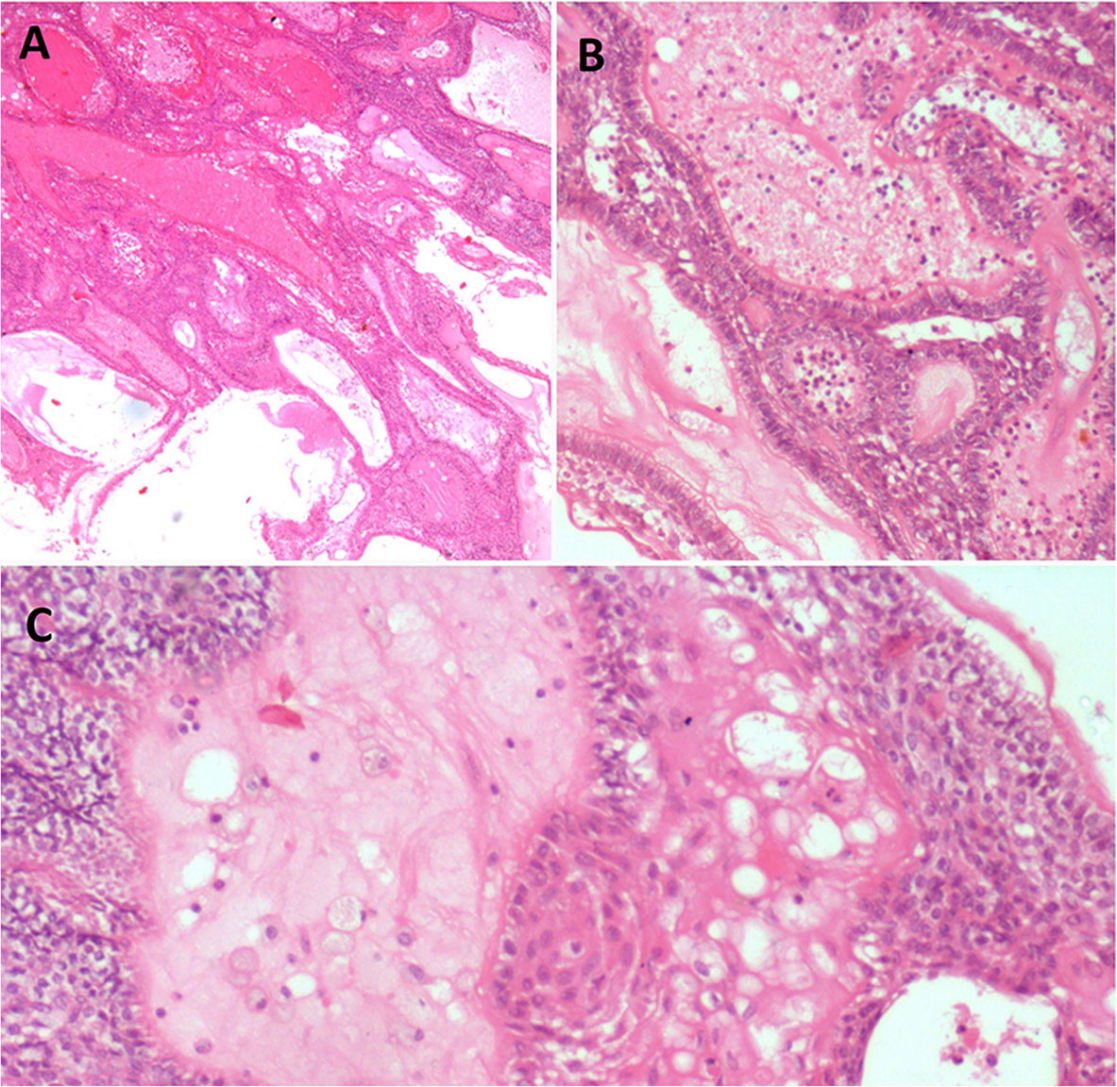
Microscopic examination of the surgical specimens. **a** Right cheek ameloblastoma. Tumoural proliferation with a follicular growth pattern of tumour islands with cystic changes and fibrous stroma. H&E × 4. **b** The tumour islands show peripheral palisading of basal columnar hyperchromatic cells with the nuclei displaced away from the basement membrane and central stellate reticulum-like cells. Detected cystic changes are also seen. H&E × 20. **c** Tumour islands with peripheral palisading and cystic changes. Areas of squamous metaplasia and keratinization are also seen (acanthomatous ameloblastoma). H&E × 20

A 75-year-old housewife, hypertensive with a previous history of right hemimandibulectomy and rib reconstruction for ameloblastoma in 1999 in plastic surgery department, MUH, presented to our centre complaining of a large cystic swelling in the floor of her mouth which recurs after aspiration that revealed haemorrhagic nature of its content. Ultrasonographic (US) examination of the neck revealed a large submental collection with internal echoes and turbid content measuring 7 × 4.5 cm. Post-contrast head and neck computed tomography (CT) examination revealed—in addition to evidence of excision of the right mandibular ramus—a thick-walled fluid collection in the floor of the mouth measuring about 6.3 × 5 × 8.2 cm with high densities inside and wall calcification (Fig. 1). It described also a well-defined partially cystic partially solid nodule in the right cheek measuring about 15 × 15 mm. Both lesions were suspected to be a recurrence. Post-contrast head and neck magnetic resonance imaging (MRI) examination described a multilocular cystic lesion in the floor of the mouth measuring 7 × 4 × 4 cm. Microscopic examination of the biopsy performed from the right buccal lesion showed tumoural proliferation formed of sheets of basaloid cells with peripheral palisading and central stellate cells surrounded by desmoplasia. This led to the diagnosis of recurrent ameloblastoma. The patient later on underwent excision of the recurrent mass en bloc with the cystic swelling through intra-oral and cervical approaches. The floor of the mouth was left intact, and the cheek defect was closed primarily. Smaller lesions were found related to the remnant of the rib and plate. They were also excised (Fig. 2). Microscopic examination of the specimens (Fig. 3) revealed a tumoural proliferation with a follicular growth pattern of tumour islands with cystic changes and fibrous stroma. The tumour islands showed peripheral palisading of basal columnar hyperchromatic cells with nuclei displaced away from the basement membrane and central stellate reticulum-like cells. Areas of squamous metaplasia and keratinization were also seen (acanthomatous ameloblastoma). Bony parts as well as safety margins were free from tumour tissue. That led to the diagnosis of recurrent ameloblastoma with free safety margins. The patient was discharged from the hospital after 5 days with an uneventful postoperative course and a very good general condition. The patient attended to the outpatient clinic for regular follow-up for 2 years, and there was neither subsequent tumour recurrence nor long-term postoperative complications.

## Discussion

Ameloblastoma is the commonest odontogenic tumour of epithelial origin with an incidence of one case per two million persons per year. Most patients’ ages range from 30 to 60 years with equal gender distribution. Despite being classified as a benign tumour, it shows locally aggressive features. It was related to inflammation, trauma and nutritional deficiencies. Yet, recent data strongly indicate that unique genetic abnormalities cause the development of ameloblastoma [1–4].

In the 2017 WHO classification of head and neck tumours, the terminology and classification of ameloblastoma were modified guided by the updates on genetic research enlightened with the studies published earlier which suggested that MAPK pathway mutations are a cornerstone in the pathogenesis of ameloblastoma. Ameloblastoma remained considered as a benign tumour despite its local aggressiveness and recurrence potentiality. The classification is now simple and limited to ameloblastoma, unicystic ameloblastoma, extraosseous ameloblastoma and malignant type. The conventional ameloblastoma is the commonest type followed by the unicystic one [1, 2, 5].

Ameloblastoma usually presents as a painless slowly growing tumour which can become a large and expansile swelling causing tooth loosening and displacement as well as facial asymmetry. CT scan is considered the gold standard for diagnosis [1, 4, 6].

Recurrence of ameloblastoma after surgical resection is not an uncommon event which reaches up to 15% even after adequate surgical resection. It can be attributed to many factors such as histological subtype, genetic characteristics of the tumour and type of surgical resection performed (conservative versus radical). It is advised to attack the primary tumour with a surgical strategy that is not so conservative to prevent recurrence and not so aggressive to prevent quality of life affection. Wide local excision with 1 to 2 cm safety margins is widely accepted. Management of recurrent ameloblastoma is a surgical challenge [1–3, 6–8]. The clinical presentation in our patient was in the form of two cystic swellings at the operative bed. CT as well as MRI was used in the preoperative evaluation. Surgical resection was challenging, and using two surgical approaches was necessary to achieve adequate resection with free margins.

Recurrence of ameloblastoma can occur in the remaining part of the mandible, surrounding soft tissue and even in the bone used for reconstruction whether in the form of graft, pedicled or free flap [6, 9]. In the presented patient, the recurrence occurred in both the operative bed and the bone used for reconstruction.

A very long time has been reported between initial presentation and recurrence exactly like our patient who had 17 years between the initial presentation and the recurrence. Essaadi et al. [7], de Miranda et al. [10] and Vasan [11] reported a patient with recurrence in the bony graft 33, 30 and 28 years, respectively, after the initial presentation.

## Conclusions

Ameloblastoma is the commonest odontogenic epithelial tumour with a local aggressive behaviour and common recurrence events despite the benign nature. Wide local excision with adequate safety margins is the most acceptable line of treatment which lowers the recurrence rates to its minimum levels. Management of recurrent ameloblastoma is challenging. Surgical excision with free margins is the best treatment modality for recurrence.

## Data Availability

All the clinical, radiological and pathological data used in this manuscript are available on the Mansoura University Medical System (Ibn Sina Hospital management system). http://srv137.mans.edu.eg/mus/newSystem/.

## Abbreviations

CT: Computed tomography
MAPK: Mitogen-activated protein kinase
MRI: Magnetic resonance imaging
US: Ultrasound
WHO: World Health Organization
